# Multidirectional flow analysis by cardiovascular magnetic resonance in aneurysm development following repair of aortic coarctation

**DOI:** 10.1186/1532-429X-10-30

**Published:** 2008-06-08

**Authors:** Alex Frydrychowicz, Raoul Arnold, Daniel Hirtler, Christian Schlensak, Aurelien F Stalder, Jürgen Hennig, Mathias Langer, Michael Markl

**Affiliations:** 1 Department of Diagnostic Radiology, Medical Physics, University Hospital Freiburg, Germany; 2 Department of Pediatric Cardiology, University Hospital Freiburg, Germany; 3 Department of Cardiovascular Surgery, University Hospital Freiburg, Germany

## Abstract

Aneurysm formation is a life-threatening complication after operative therapy in coarctation. The identification of patients at risk for the development of such secondary pathologies is of high interest and requires a detailed understanding of the link between vascular malformation and altered hemodynamics. The routine morphometric follow-up by magnetic resonance angiography is a well-established technique. However, the intrinsic sensitivity of magnetic resonance (MR) towards motion offers the possibility to additionally investigate hemodynamic consequences of morphological changes of the aorta.

We demonstrate two cases of aneurysm formation 13 and 35 years after coarctation surgery based on a Waldhausen repair with a subclavian patch and a Vosschulte repair with a Dacron patch, respectively. Comprehensive flow visualization by cardiovascular MR (CMR) was performed using a flow-sensitive, 3-dimensional, and 3-directional time-resolved gradient echo sequence at 3T. Subsequent analysis included the calculation of a phase contrast MR angiography and color-coded streamline and particle trace 3D visualization. Additional quantitative evaluation provided regional physiological information on blood flow and derived vessel wall parameters such as wall shear stress and oscillatory shear index.

The results highlight the individual 3D blood-flow patterns associated with the different vascular pathologies following repair of aortic coarctation. In addition to known factors predisposing for aneurysm formation after surgical repair of coarctation these findings indicate the importance of flow sensitive CMR to follow up hemodynamic changes with respect to the development of vascular disease.

## Introduction

Despite advances in endovascular therapy, operative procedures still play a major role in the treatment of aortic coarctation. In the newborn, a severe coarctation is a life-threatening finding and immediate invasive therapy is the method of choice [1]. As a consequence, there are large numbers of patients who have survived treatment in the past and are at risk of developing secondary complications induced by vascular remodeling and impaired vascular reactivity. Common secondary pathologies are increased intima-media thickness, aortic arch hypoplasia, restenosis, and aneurysm formation decades after surgery [2-5]. Aneurysms may develop at the site of the aortic repair which is most likely associated with the operative technique. In this context, the largely discrepant compliance of the materials used for the patch plastic and the associated gradients along the vessel wall as well as local dehiscence within the suture have been discussed [6-9]. The associated complication rate may be seen as the driving force as to why the Dacron patch plastic (Vosschulte repair) has been more and more replaced by the subclavian patch angioplasty (Waldhausen repair) and a direct end-to-end anastomosis after surgical excision of the stenosis.

Aneurysms developing further upstream in the ascending aorta may be related to co-morbidities such as bicuspid aortic valve, high perioperative blood pressure, the operative procedure [10], and the patient's age at the initial repair [2]. However, little is known about the underlying process leading to such vascular changes. Although altered hemodynamics may not be the driving force their contribution, e.g. as a mediating mechanism, can not be neglected [5] and may have an impact on secondary complications such as poststenotic dilatation.

Furthermore, the identification of patients at risk for the development of such secondary pathologies would be best served by a detailed understanding of the inter-relationship between vascular pathology and the role of altered hemodynamics which is currently not well understood. In this context, regular follow-up examinations are necessary and contrast enhanced magnetic resonance (MR) angiography is increasingly used as a standard non-invasive method in the evaluation of coarctation of the aorta [11,12]. Moreover, the ability of cardiovascular MR (CMR) to measure blood flow in-vivo and recent methodological developments have provided novel diagnostic tools that permit the direct assessment and analysis of 3D blood flow in the heart or large and mid-sized vessels [13-17].

In this study, a comprehensive 4D CMR protocol was employed which permits the simultaneous acquisition of vascular geometry and associated 3D hemodynamics in-vivo. Findings in two patients illustrate marked changes in 3D blood flow 13 and 35 years after aortic surgery in differently shaped aneurysms after Waldhausen & Nahrwold [18] and Vossschulte [19,20] repair, respectively.

## Methods

The CMR protocol included 3-dimensional, 3-directional, time-resolved phase contrast velocity mapping ("comprehensive CMR velocity acquisitions"), based on a radio frequency spoiled gradient echo sequence using a 3 Tesla CMR system (Magnetom TRIO, Siemens, Erlangen, Germany).

Cardiac and navigator gating was performed to permit ECG synchronized measurement of 3-directional blood flow in the entire thoracic aorta during free breathing. Imaging parameters were adjusted to the individual patient and were as follows: flip angle = 15°, velocity sensitivity = 150 cm/s, spatial resolution = 2.5 × 1.8 × 2.6 mm^3 ^(patient 1)/3.0 × 1.6 × 3.2 mm^3 ^(patient 2), temporal resolution = 40.0 ms (patient 1)/48.8 ms (patient 2). Written informed consent was obtained form all subjects after approval of the local ethics committee. Contrast agent was applied for the routine diagnostic follow-up examination including a contrast-enhanced MR-angiography (CE-MRA) in patient 1 only.

Data analysis included the calculation 3D phase contrast angiograms (PC-MRA) from the comprehensive CMR velocity acquisitions data and 3D visualization of the measured time-resolved three-directional blood flow velocities (EnSight, CEI, Apex, NC). PC-MRA data were visualized as gray or red shaded iso-surfaces, respectively. Flow visualization included the calculation of time-resolved 3D particle traces and 3D stream lines [21-23]. Time-integrated particle traces, i.e. the paths of virtual particles released within measured flow velocity field, were used to summarize the aspects of the temporal evolution of blood flow over the cardiac cycle within a single image.

**Figure 1:**
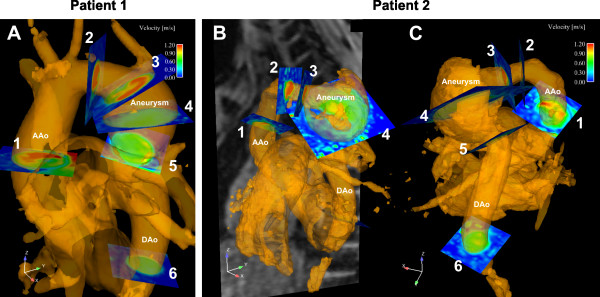
Cutplane locations in patient 1 (A) and 2 (B, C) for quantitative analysis of flow and wall parameters. Cutplanes 3–5 were placed at the proximal and distal neck of the aneurysm (3 and 5, respectively) whereas cutplane 4 was positioned such that the middle of the aneurysm was transected, i.e. the plane was oriented orthogonal to the projected direct blood flow form proximal to distal neck.

For quantitative analysis, 2D cutplanes were manually positioned in the ascending aorta, transverse aortic arch, proximal and distal neck as well as middle of the aneurysm, and the descending aorta (see figure 1). Next, multidirectional velocity data in these planes were extracted and evaluated with a home built tool (Matlab, USA). For each cutplane analysis included manual frame-wise lumen segmentation and calculation of wall shear stress (WSS), oscillatory shear index (OSI), peak mean (Vel_max_) and time-averaged mean (Vel_mean_) absolute velocities, and vessel diameter as described previously [24]. Note that WSS, i.e. the spatial velocity gradient at the vessel wall, is a time-resolved vector quantity and can be fully derived from the measured multidirectional velocity fields. For each cutplane WSS values were averaged over the cardiac cycle and along the lumen contour. To account for the vector nature of WSS the time-averaged magnitude (WSS_mag_) as well as axial (WSS_ax_) and circumferential (WSS_circ_) components are given. OSI was calculated based on WSS_mag _according to previously reported strategies by He and Ku [25].

## Results

### Case reports

The first patient (age = 19 years, male) was scheduled for regular follow-up MRA 13 years after aortic reconstruction by a Waldhausen and Nahrwold subclavian flap angioplasty of the stenotic site. Over the past four years, contrast-enhanced MRA revealed progression of a postoperatively developing concentric true aneurysm with a maximum diameter increasing from 3.6 to 4.2 cm. Due to the rapid progress the patient was scheduled for the placement of an endovascular covered stent (Medtronic Valiant Thoracic 28/32 × 150) with a carotis-subclavian bypass and was sent for additional hemodynamic analysis before. The combined operative and endovascular procedure was successful; the patient recovered rapidly and was discharged from hospital shortly thereafter.

The second patient (age = 37 years, male) was examined 35 years after a Vossschulte approach resecting the stenotic site and performing a Dacron patch plastic. The patient did not undergo regular angiographic follow-up and a large eccentric aneurysm of 6.8 cm diameter was diagnosed coincidentally by digital subtraction angiography in the workup of new onset of shortness of breath and an episode of hemoptysis. Before the patient underwent surgical repair by aneurysm resection and interposition of a 22 mm Dacron graft he was also sent for hemodynamic evaluation. During surgery, the eccentric true aneurysm revealed a thinned anterior wall on which the patch was centered and a normal posterior vessel wall. The patient recovered from surgery without complications and was discharged quickly after surgery. During follow-up, no complications were encountered; the patient tolerated physical stress well.

### Morphometric and hemodynamic analysis

**Figure 2:**
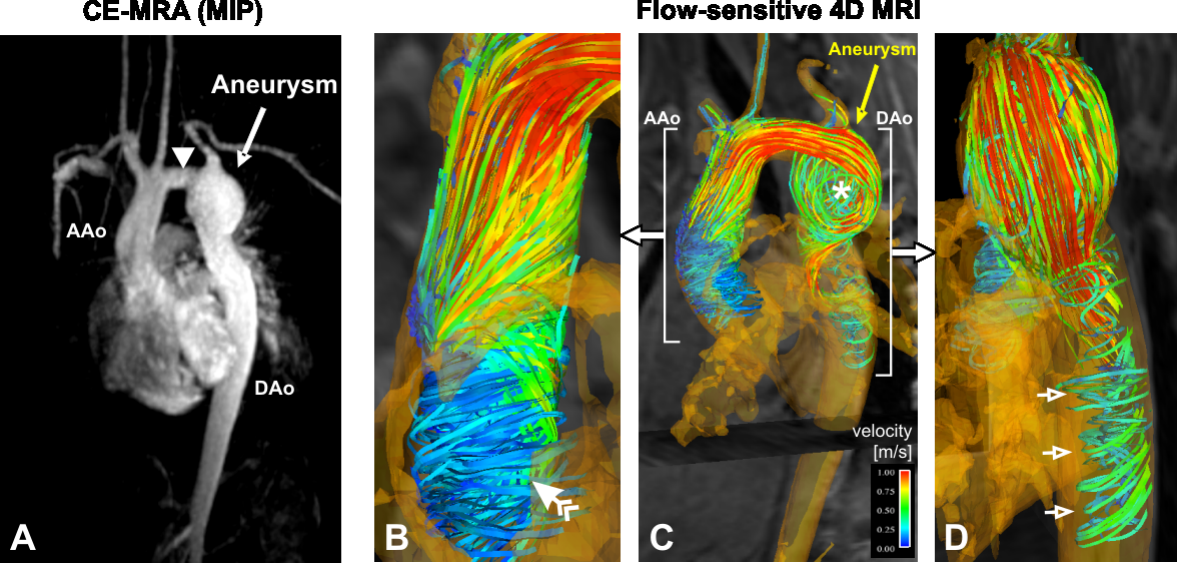
Findings in patient 1, 13 years after operative repair of aortic coarctation. **A**: contrast enhanced MRA with a tubular shaped transverse arch (white arrow tip) and an aneurysm of the proximal descending aorta with a diameter of 4.2 cm. **B-D**: 3D visualization of blood flow measurements using time-integrated particle traces. Note three obvious findings. In C (lateral view) blood flow acceleration and a slow vortical flow through the aneurysm with the centre of the flow vortex at the lateral wall (*) can be appreciated. B (anterior view) and D (posterior view) show pronounced helical flow in the ascending aorta (AAo, feathered arrow) and circular downstream flow in the descending aorta (DAo, open white arrows). A more detailed depiction of the temporal evolution of 3D blood flow is provided in the additional file 1.

**Figure 4:**
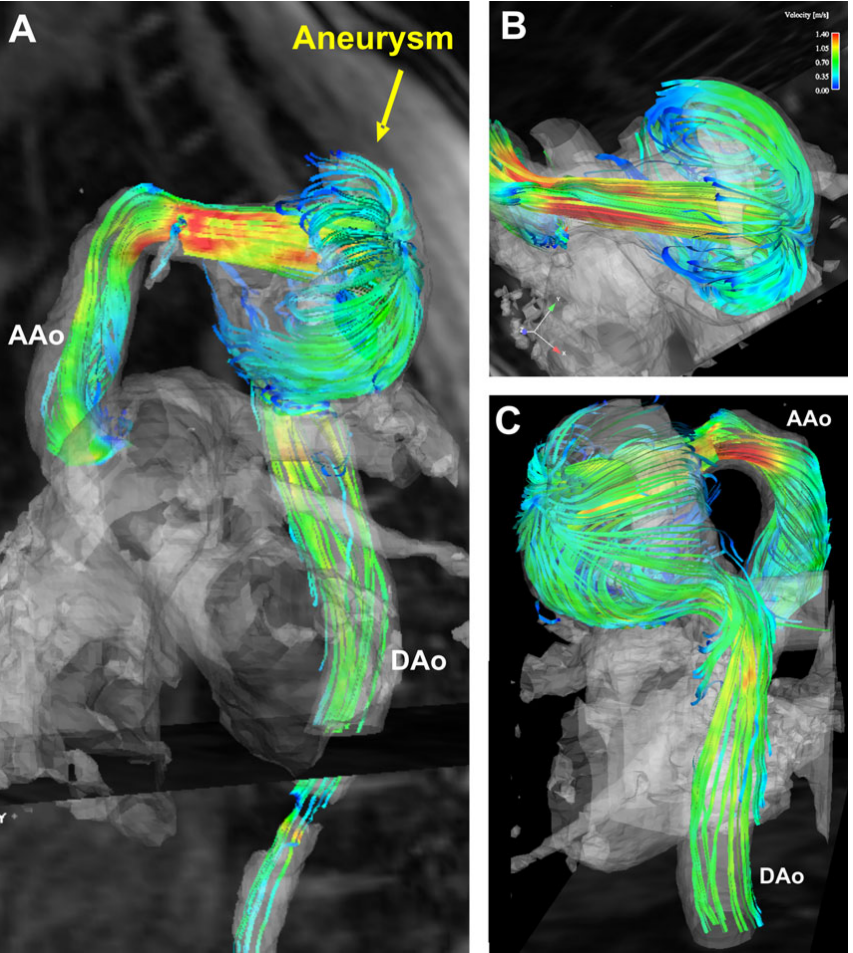
3D stream-lines representing vascular hemodynamics in a 37 year-old male 35 years after surgical repair of aortic coarctation. Note the flow acceleration through the tubular shaped transverse arch inducing a flow jet into the aneurysm and circumferential separation and recirculation of the inflowing streamlines which develop into an umbrella-like blood flow pattern. A = lateral view, B = cranial view, C = posterior view. A more detailed depiction of the temporal evolution of 3D blood flow is provided in the additional file 2.

Both patients presented with near normal diameters of the ascending aorta and transverse arch although the arch presented with reduced lumen diameters (1.9 cm and 2.3 cm) and a tube-like shape directly pointing towards the aneurysms in the proximal descending aorta. For a listing of quantitative findings please refer to table 1 and see figures 2 and 4)

**Table 1:** Listing of quantitative parameters in both patients.

	**CASE 1**						
	
	**Diam. [cm]**	**Vel_mean _[m/s]**	**Vel_max _[m/s]**	**WSS_mag _[N/m^2^]**	**WSS_ax _[N/m^2^]**	**WSS_circ _[N/m^2^]**	**OSI [%]**
**AAo**	3.2	0.18	0.62	0.17	0.14	0.10	17.4
**Transverse arch**	**2.3**	**0.20**	**0.77**	**0.23**	**0.22**	**0.08**	**10.4**
**DAo**	3.0	0.08	0.36	0.13	0.11	0.07	16.8
**Prox. Aneurysm neck**	2.8	0.11	0.5	0.13	0.12	0.06	23.9
**Aneurysm**	**4.7**	**0.03**	**0.18**	**0.14**	**0.11**	**0.08**	**19.4**
**Dist. Aneurysm neck**	3.0	0.11	0.44	0.26	0.10	0.24	12.0

	**CASE 2**						

**AAo**	2.6	0.19	0.58	0.29	0.24	0.17	12.5
**Transverse arch**	**1.9**	**0.22**	**0.69**	**0.24**	**0.21**	**0.11**	**14.6**
**DAo**	2.5	0.19	0.45	0.22	0.19	0.12	21.5
**Prox. Aneurysm neck**	2.2	0.15	0.48	0.14	0.11	0.09	19.8
**Aneurysm**	**6.2**	**0.04**	**0.08**	**0.15**	**0.07**	**0.13**	**24.9**
**Dist. Aneurysm neck**	2.5	0.16	0.40	0.19	0.16	0.11	19.8
	
	**MEAN WSS BOTH CASES**			**<WSS**_mag_**> [N/m^2^]**	**<WSS**_ax_**> [N/m**^2^**]**	**<WSS**_circ_**> [N/m^2^]**	**<OSI> [%]**
	
	**Average WSS AAo**			0.23	0.19	0.14	15.0
	**Average WSS Arch**			0.24	0.22	0.10	12.5
	**Average WSS Aneurysm**			0.17	0.11	0.12	20.0

Average and peak velocities are increased in the traverse tubular arch and substantially reduced inside the aneurysm. In both cases absolute (WSS_mag_) and in particularly axial (WSS_ax_) wall shear stress are lower at the aneurysm wall compare to ascending aorta and transverse arch. Note that circumferential wall shear stress (WSS_circ_) in the aneurysm remains high or is regionally even increases most likely related to enhanced vortex or helix flow in the aneurysm. AAo = Ascending aorta, Dao = descending aorta, WSS = wall shear stress, OSI = oscillatory shear index, Diam = diameter, Vel = velocity.

**Figure 3:**
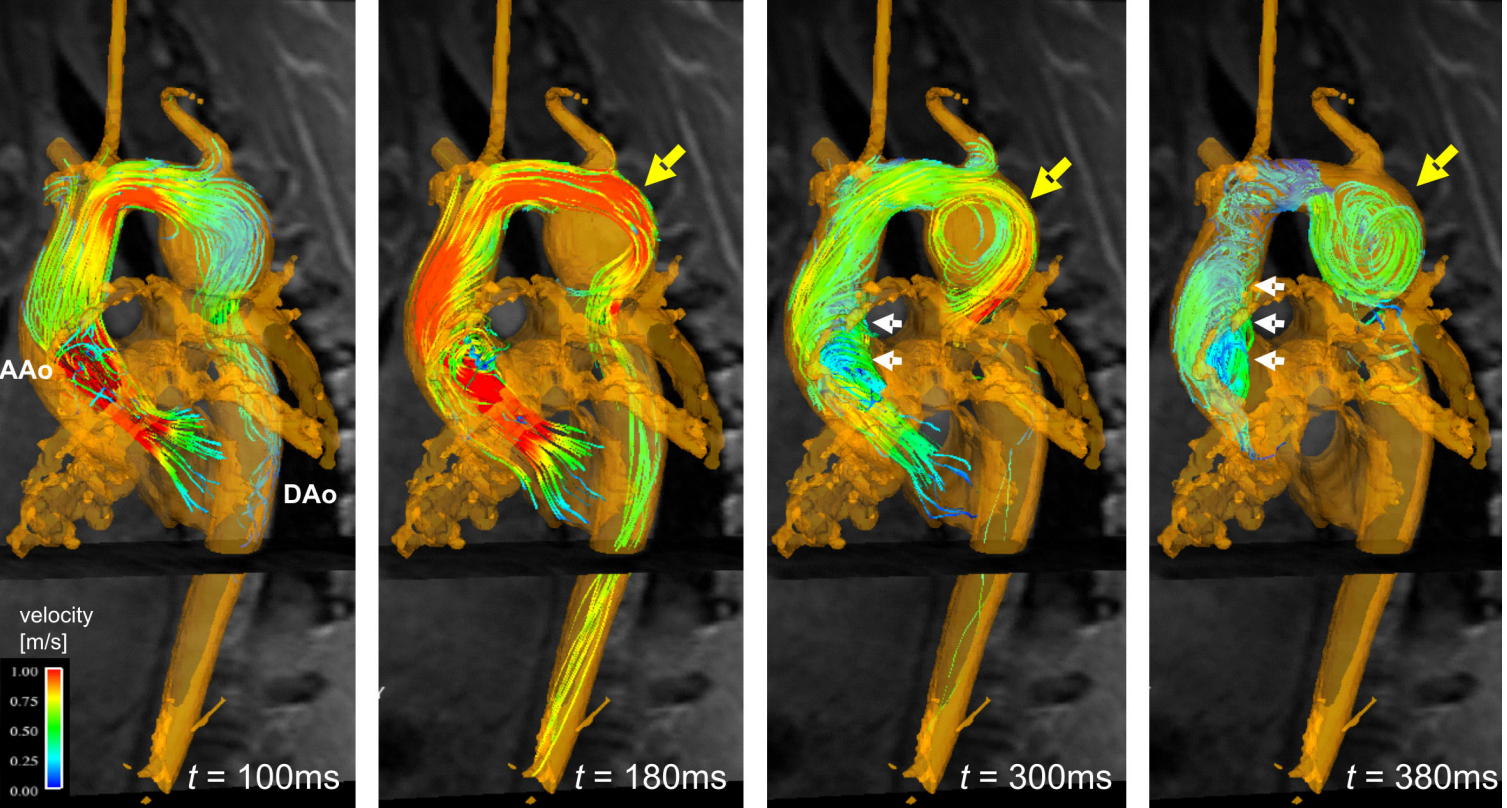
3D flow pattern development in the thoracic aorta in patient 1 with a tubular shaped aortic arch and an aneurysm of the proximal descending aorta (yellow, arrow, diameter = 4.2 cm). 3D stream-lines within the 3D-PC-MRA iso-surface illustrate accelerated flow along the outer aneurysm wall (t = 180 ms) and subsequent formation of a flow vortex (t = 300 ms and t = 380 ms). Note that aneurysm formation affects blood flow in the entire aorta resulting in marked helical flow in the ascending aorta (AAo, white arrows).

Comprehensive CMR velocity acquisitions for the simultaneous assessment of vascular geometry and time-resolved 3D blood flow in the entire thoracic aorta confirmed these findings and revealed marked and differing changes in 3D blood flow. In both patients, the 3D visualization of the measured blood flow velocities revealed pronounced flow acceleration within the tubular transverse arch segment leading to a flow yet directed towards the orifice of the aneurysm (figures 2, 3, and 4). These qualitative findings were confirmed by increased mean and maximum velocities in the respecting cutplane positioned in the transverse arch for both patients (see tab. 1).

The aneurysmal blood flow patterns, however, demonstrated distinct differences between patients. In patient 1, the flow jet developed into a swirling circular pattern which likely results from the tangential inflow with respect to the cavity. Further, a separation and recirculation of inflowing streamlines inferiorly more than superiorly can be appreciated (see figures 2, 3 and the additional file 1). Time-resolved flow patterns in figure 4 clearly illustrate the conversion of the initial flow acceleration along the outer curvature into subsequent formation of largely vortical and recirculating flow within the aneurysm that implies a loss of kinetic energy and momentum. In patient 2, in which the cavity extends superiorly and bilaterally as well as inferiorly with respect to the transverse arch, flow acceleration within the aortic arch presents a circumferential separation and recirculation of the inflowing streamlines which expands into an umbrella-shaped flow pattern with a point of maximum impact located at the craniolateral wall of the aneurysm wall (see figure 4 and the additional file 2).

Along with the marked reduction of mean and maximum blood flow velocities within the aneurysm, vectorial WSS and OSI calculations revealed interesting findings (see table 1). Average and peak velocities are increased in the traverse tubular arch and substantially reduced inside the aneurysm. In both cases absolute (WSS_mag_) and in particularly axial (WSS_ax_) wall shear stress are lower at the aneurysm wall compare to ascending aorta and transverse arch which are in the order of previously published results [26]. Note that circumferential wall shear stress (WSS_circ_) in the aneurysm remained high or was regionally even increases indicating increased flow directed along the vessel lumen circumference. In addition, the OSI underlines what seems obvious from the visual analysis and demonstrated higher values within the aneurysm.

## Discussion

Findings of these exemplary patient data demonstrate the diversity in hemodynamics associated with aneurysm formation after surgery in aortic coarctation. The results in two cases represent different shapes of true aneurysms and the associated blood flow patterns.

Although the cause for secondary aneurysm formation after repair of coarctation is most likely multifactorial and can not only be found in the hemodynamics they may in turn be associated with aneurysm formation or play a mediating or enhancing role. From the literature it is known that unfavorable shear stress can create areas at risk for vascular remodeling [3,27,28]. Such hemodynamic changes may thus facilitate aneurysm formation or promote its development if predisposing factors such as stress gradients along the vascular wall with implanted patches or sutures are present.

As shown for the exemplary concentric aneurysm (patient 1), a relative flow acceleration in the aortic arch developed into a flow pattern adapted to the shape of the aneurysm, i.e. highly circulating flow with a vortex core near the lateral wall. No link between both aneurysm shapes should be concluded such that eccentric aneurysms necessarily develop from concentric forms. However, the eccentrically shaped aneurysm presents with far more pronounced flow alterations and exaggerated circumferential separation and recirculation of the inflowing streamlines. These findings are further supported by the result that OSI and the fraction of circumferential WSS remains high or is increased whereas the axial WSS magnitude was considerably reduced. Whether these factors may prove useful for prediction of the severity of the disease remains speculative and is subject to larger patient studies. Note however, that in these two cases major WSS contribution were associated with the circumferential component indicating the importance to consider the vector nature of WSS.

A potential limitation of the methodology can be seen in the fact that the underlying velocity data is acquired over many cardiac cycles and effectively averages cyclic variations of flow. Hence, small scale instabilities or turbulence in blood flow properties may not be depicted. However, the presented comprehensive CMR velocity acquisitions, visualization strategy, and quantification possibilities are promising to further improve the understanding of vascular pathologies and associated hemodynamics as well as comprehensively assess cardiovascular physiology in-vivo by CMR.

## Competing interests

The authors declare that they have no competing interests.

## Authors' contributions

AF, RA, CS and MM were responsible for patient recruitment, all measurements and data evaluation, DH and AFS performed 3D flow visualization and quantitative data analysis, JH and ML participated in the design of the study and assisted in data evaluation and interpretation, MM conceived of the study, and participated in its design and coordination. All authors read and approved the final manuscript.

## Supplementary Material

Additional file 1 Time-resolved 3D particle traces in patient 1, 13 years after operative repair of aortic coarctation by a Waldhausen subclavian flap angioplasty. The movie illustrates the temporal evolution of blood flow originating from emitter planes in the ascending aorta and aortic arch. Three distinct flow patterns can clearly be appreciated: Accelerated flow in the tubular shaped aortic arch, vortex formation within the descending aortic aneurysm, and considerable helical flow in the ascending and descending aorta. Color coding = local blood flow velocity magnitude. Click here for file

Additional file 2 Time-resolved particle traces in patient 2, 35 years after coarctation repair by Vossschulte approach. Clearly, the increased velocities in the hypoplastic aortic arch can be appreciated. The blood flow is accelerated towards the opposite wall of the eccentric aneurysm, presents a circumferential separation and recirculation of the inflowing streamlines and is redirected in all directions in an umbrella-like fashion. Click here for file
